# Notch1 promotes resistance to cisplatin by up-regulating Ecto-5′-nucleotidase (CD73) in triple-negative breast cancer cells

**DOI:** 10.1038/s41420-023-01487-x

**Published:** 2023-06-30

**Authors:** Yuzhu Qi, Meifang Li, Shaozhong Li, De Zeng, Yingsheng Xiao, Jiwei Li, Qianqian Ye, Edwin Bremer, Guo-jun Zhang

**Affiliations:** 1 Cancer Center & Department of Breast and Thyroid Surgery, Xiang’an Hospital of Xiamen University, School of Medicine, Xiamen University, 361101 Xiamen, China; 2 Department Of Hematology, University Medical Center Groningen, University of Groningen, Groningen, The Netherlands; 3 Department of Medical Oncology, Cancer Hospital of Shantou University Medical College, Shantou, China; 4 The first affiliated hospital of Gannan Medical University, Ganzhou, Jiangxi Province China; 5 Shenshan Medical Center, Sun Yat-sen Memorial Hospital, Sun Yat-sen University, 516621 Shanwei, China; 6 Department of Thyroid Surgery, Shantou Central Hospital, Shantou, China; 7 Department of Respiratory, Critical Care and Sleep Medicine, Xiang’an Hospital of Xiamen University, School of Medicine, Xiamen University, Xiamen, China; 8 Department of Pathology, Maternal and Child Health Hospital of Ganzhou, Ganzhou, Jiangxi China; 9 Fujian Key Laboratory of Precision Diagnosis and Treatment in Breast Cancer (Xiang’an Hospital of Xiamen University), 361101 Xiamen, China; 10 Xiamen Key Laboratory of Endocrine-Related Cancer Precision Medicine, Xiang’an Hospital of Xiamen University, 361101 Xiamen, China; 11 Xiamen Research Center of Clinical Medicine in Breast & Thyroid Cancers, Xiang’an Hospital of Xiamen University, 361101 Xiamen, China; 12 Central Laboratory, Xiang’an Hospital of Xiamen University, 361101 Xiamen, China

**Keywords:** Breast cancer, Chemotherapy, Breast cancer

## Abstract

Triple-negative breast cancer (TNBC) is an aggressive molecular subtype that due to lack of druggable targets is treated with chemotherapy as standard of care. However, TNBC is prone to chemoresistance and associates with poor survival. The aim of this study was to explore the molecular mechanisms of chemoresistance in TNBC. Firstly, we found that the mRNA expression of Notch1 and CD73 in cisplatin-treated patient material associated with poor clinical outcome. Further, both were upregulated at the protein level in cisplatin-resistant TNBC cell lines. Overexpression of Notch1 intracellular domain (termed N1ICD) increased expression of CD73, whereas knockdown of Notch1 decreased CD73 expression. Using chromatin immunoprecipitation and Dual-Luciferase assay it was identified that N1ICD directly bound the CD73 promoter and activated transcription. Taken together, these findings suggest CD73 as a direct downstream target of Notch1, providing an additional layer to the mechanisms underlying Notch1-mediated cisplatin resistance in TNBC.

## Introduction

Triple-negative breast cancer (TNBC) is characterized by lack of expression of the estrogen receptor (ER), the progesterone receptor (PR) and HER-2 [1]. Compared to other breast cancer subtypes, TNBC is more prone to formation of regional or distant metastases and associates with a worse prognosis [2]. Due to a lack of specific molecular targets the main systemic treatment for TNBC remains cytotoxic chemotherapy, including adriamycin and taxane or platinum-based chemotherapy [3, 4].

TNBC is relatively sensitive to DNA damaging agents [5], such as platinum-based drugs that induce DNA cross-linking [6]. Indeed, platinum-based regimens yield higher pathologic complete remission rates (pCR) in neoadjuvant setting than other regimens [7]. A phase III trial further demonstrated that carboplatin doubled the objective response rate compared to docetaxel treatment (68% versus 33%) in subjects with germline-mutated BRCA 1/2 breast cancer, suggesting that a platinum-based regimen is more effective for TNBC patients [8]. However, many patients develop resistance to chemotherapy and relapse after treatment [9]. In order to improve treatment options for TNBC, it is therefore critical to gain understanding of the mechanism of chemoresistance.

A protein of particular interest in this respect is Notch1 (termed N1), belonging to the highly conserved Notch family of transmembrane proteins critical for organ development [10]. Notch signaling is induced by the process of regulated intramembrane proteolysis (RIP), which yields the Notch intracellular domain (NICD) that translocates to the nucleus. In the nucleus, the NICD binds to the DNA-binding protein CSL (CBF-1, suppressor of hairless, LAG-1), thereby, releasing transcriptional repression of target genes [11]. The Notch signaling pathway has previously been shown to be involved in tumorigenesis and progression of breast cancers [12]. Moreover, increasing evidence links Notch1 to chemoresistance through upregulation of ABC transporters [13], with e.g., N1ICD binding to the ABCC1 promoter to upregulate expression. Further, Notch1 regulates expression of apoptotic proteins [14], augments the cancer stem cell phenotype [15] and can activate the p53 pathway [16]. Notch1 signalling further was shown to contribute to cisplatin resistance through various downstream targets, including major vault protein [17], CD146 [18], Hes1 [19], and Jagg1 [20].

CD73, or Ecto-5′-nucleotidase, is expressed on the cell surface of various cells and has recently been linked to poor prognosis in breast cancer [21]. Specifically, CD73 expression was elevated in tumor lesions compared to surrounding normal tissue, and promoted tumor angiogenesis, invasion and metastasis in a xenograft model of TNBC. Furthermore, CD73 expression increased the resistance of TNBC to chemotherapy, including to treatment with adriamycin [22], carboplatin, gemcitabine, and paclitaxel [23].

In the present study, we identified CD73 as a direct downstream target of Notch1, with CD73 expression promoting resistance to cisplatin in triple-negative breast cancer cells.

## Results

### Notch1 and CD73 are elevated in a model of acquired cisplatin resistance in TNBC and correlate with cisplatin resistance

**Fig. 1:**
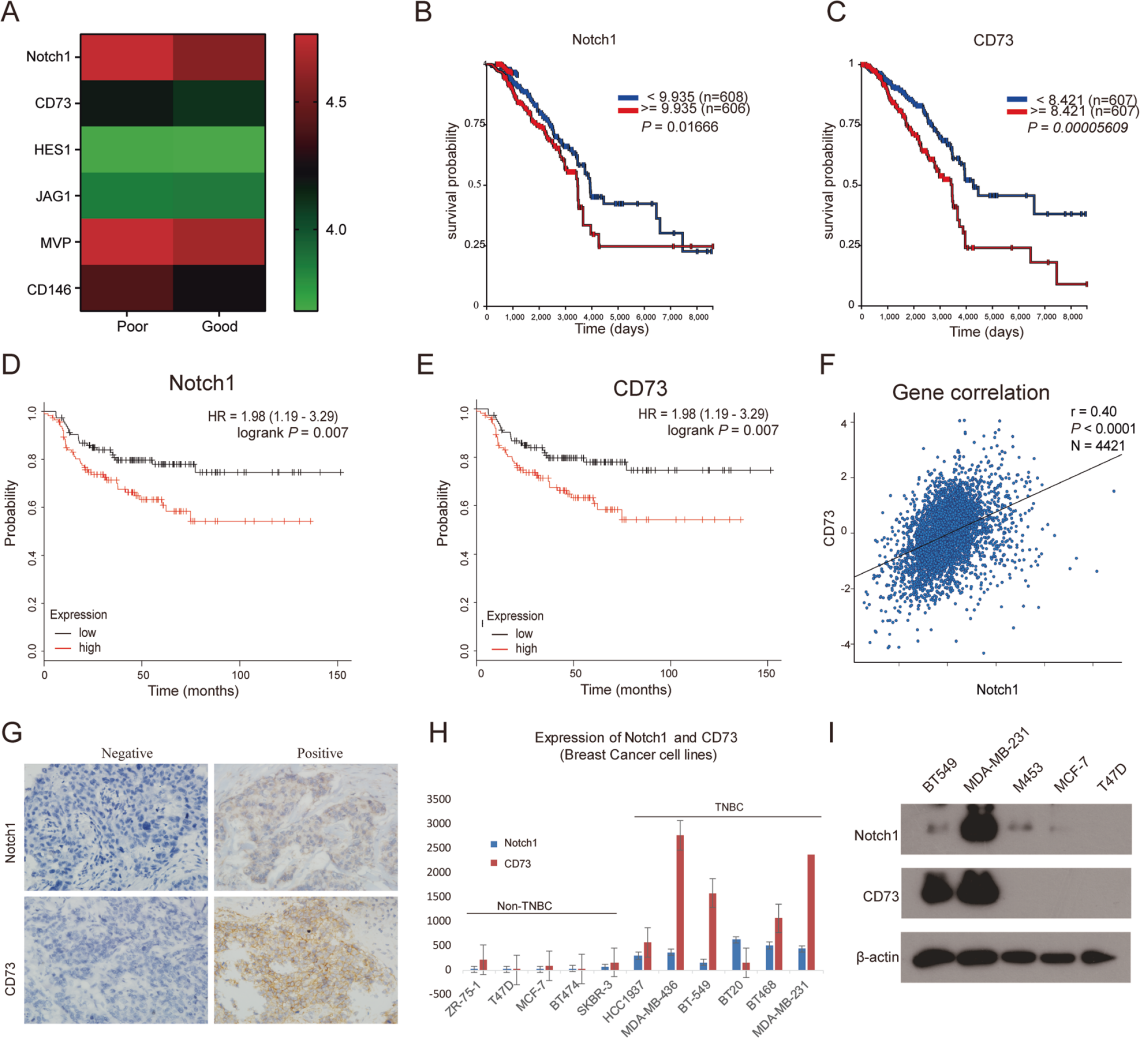
Notch1 and CD73 are highly expressed in worse cisplatin response model and have a positive correlation. **A** Heatmap of differentially expressed genes in breast cancer patients who have poor pathological response group (poor) or good pathological response (good) to cisplatin. **B**, **C** overall survival analysis of high and low expression of Notch1 and CD73 in patients with breast cancer respectively. **D**, **E** RFS analysis of Notch1 and CD73 expression level in basal-like breast cancer patients with adjuvant chemotherapy. **F** Gene correlation targeted analysis between Notch1 and CD73 (*N* = 4421). **G** Immunohistochemical pictures of Notch1 and CD73 in TNBC patients’ tissues. **H** Notch1 and CD73 mRNA expression levels analysis in various breast cancer cell lines in GEO (GSE12777). **I** Notch1 and CD73 protein expression levels in various primary breast cancer cell lines.

To evaluate expression and a possible link between Notch1 and CD73, a heatmap of publicly available gene expression data was generated from clinical trials with single-agent treatment with cisplatin (GSE18864) (Fig. 1A). Notch1 and CD73 mRNA expression levels were higher in the poor pathological response group. Further, higher Notch1 and CD73 expression associated with poor overall survival in a set of 1247 breast cancer patients (Fig. 1B, C). In addition, high level of Notch1 mRNA predicted for poor recurrence-free survival (RFS) (*n* = 230, HR = 1.81, *p* = 0.0199) in basal-like BC patients with chemotherapy (Fig. 1D), as well as ER + BC parients (Supplement 1A,B) and Her2+ BC patients (Supplement 1C,D), with mRNA expression of CD73 also correlating with poor RFS (*n* = 230, HR = 1.98, *p* = 0.007) (Fig. 1E). These data link both high notch1 and CD73 expression levels to poor prognosis of TNBC patients.

In a further analysis of 4421 breast tumor patients, expression levels of CD73 correlated with the mRNA expression level of Notch1 (Fig. 1F, *r* = 0.40, *p* < 0.001). Upon subsequent immunohistochemical assessment of 53 TNBC samples, expression of both Notch1 and CD73 were clearly detected (Fig. 1G), with a positive correlation between expression of both proteins (Pearson = 0.33, *p* = 0.016) (Table 2). In an online mRNA expression profiling array of human cell lines, Notch1 and CD73 were also significantly elevated in TNBC subgroups compared to non-TNBC cell lines (GSE12777) (Fig. 1H). In addition, protein levels of Notch1 and CD73 were significantly higher in TNBC cells (BT549, MDA-MB-231, and M453) than in non-TNBC breast cancer cells (T47D and MCF-7) (Fig. 1I). Expression levels of both Notch1 and CD73 in BC cell lines were verified in an online database of sequenced patient samples (bc-GenExMiner v4.8), the mRNA level of Notch1 and CD73 in TNBC subtype tumors was significantly higher than non-TNBC subtype counterparts (Supplement 1E,F).

### Notch1 directly regulated the expression level of CD73 in cisplatin-resistant cells and primary TNBC cell lines

**Fig. 2:**
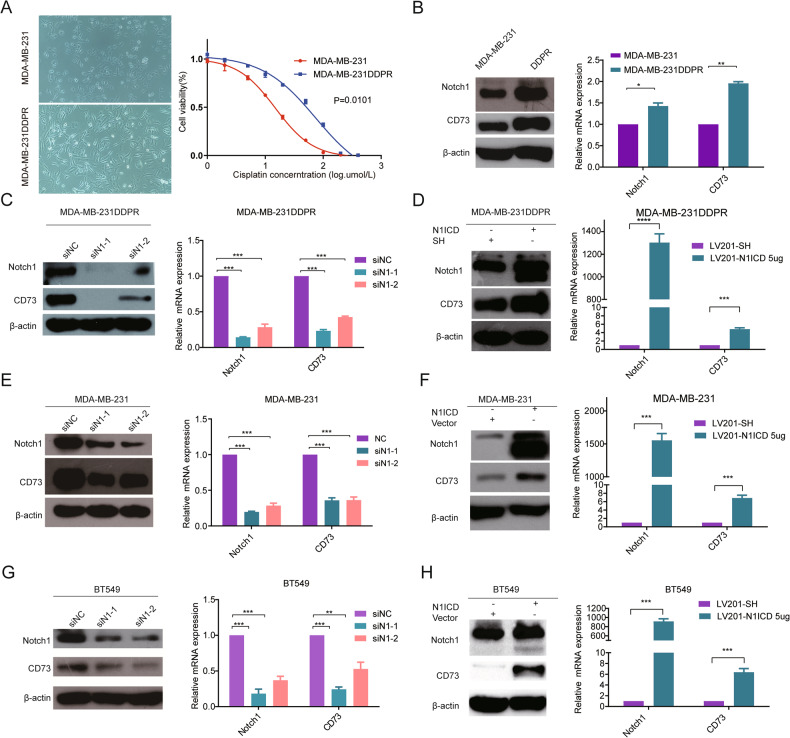
Positive correlation of Notch1 and CD73 expression in breast cancer cell lines. **A** Cellular morphology of MDA-MB-231 cells (Up) and MDA-MB-231DDPR cells (Down). **B** Cell viability analysis of MDA-MB-231 and MDA-MB-231DDPR cells using CCK-8 assay after treatment with cisplatin. **C** and **D** Real-time RT-PCR and Western blot detected that, ectopically over-expressed Notch1 Intracellular Domain (N1ICD) or Notch1 knockdown, the Notch1 and CD73 expression in cisplatin-resistant cells respectively. **E** and **F** The relative mRNA expressions and the protein levels of Notch1 and CD73 were determined, following ectopically over-expressed N1ICD in a dose-dependent way or simultaneously transfected with two siRNA targeting Notch1, by quantitative real-time PCR and western blot in MDA-MB-231cells. And (**G**) and (**H**) in BT549. Data were presented as the mean ± SEM in three independent experiments. *p < 0.05, **p < 0.01 and ***p < 0.001 (Student’s *t* test) as compared with control cells.

To evaluate whether Notch1 and CD73 expression also associated with cisplatin resistance in vitro, a cisplatin-resistant cell line (MDA-MB-231DDPR) was established with an IC50 ~ 5-fold higher at 74.58 ± 1.299 (umol/L) than that of MDA-MB-231 wild-type cells 14.95 ± 1.029 (umol/L) (Fig. 2A). In line with expectations, the expression of Notch1 and CD73 was significantly upregulated at the mRNA and protein level in MDA-MB-231DDPR compared to wild-type cells (Fig. 2B). Taken together, Notch1 and CD73 expression may associate with poor chemotherapeutic response in breast cancer.

In order to clarify the relationship between Notch1 and CD73 expression in cisplatin resistant MDA-MB-231DDPR cells, Notch1 was knocked down using two different siRNAs, resulting in an ~70% downregulation of Notch1 mRNA levels in both cases, with siN1-1 yielding the strongest downregulation (Fig. 2C). Notably, Notch1 siRNA treatment also significantly reduced mRNA expression of CD73 to a similar extent, again with siN1-1 yielding the strongest effect. This downregulation at the mRNA level was mirrored by the downregulation of both Notch1 and CD73 at the protein level as determined by western blot aanlysis, with prominent downregulation of both Notch1 and CD73 upon Notch1 siRNA treatment. Reversely, transient overexpression of the intracellular domain of Notch1 (termed N1ICD) in MDA-MB-231DDPR cells significantly upregulated not only Notch1 expression but also expression of CD73 at the mRNA and protein level (Fig. 2D).

In parental cisplatin sensitive MDA-MB-231 cells, the expression of CD73 was also decreased significantly when Notch1 was knocked-down (Fig. 2E). In contrast, CD73 expression was greatly upregulated following overexpression of Notch1 (Fig. 2F). Similarly, Notch1 siRNA treatment resulted in a 50% reduction in CD73 expression in BT549 cells (Fig. 2G), whereas Notch1 overexpression resulted in a 6–9-fold increase (Fig. 2H).

**Fig. 3:**
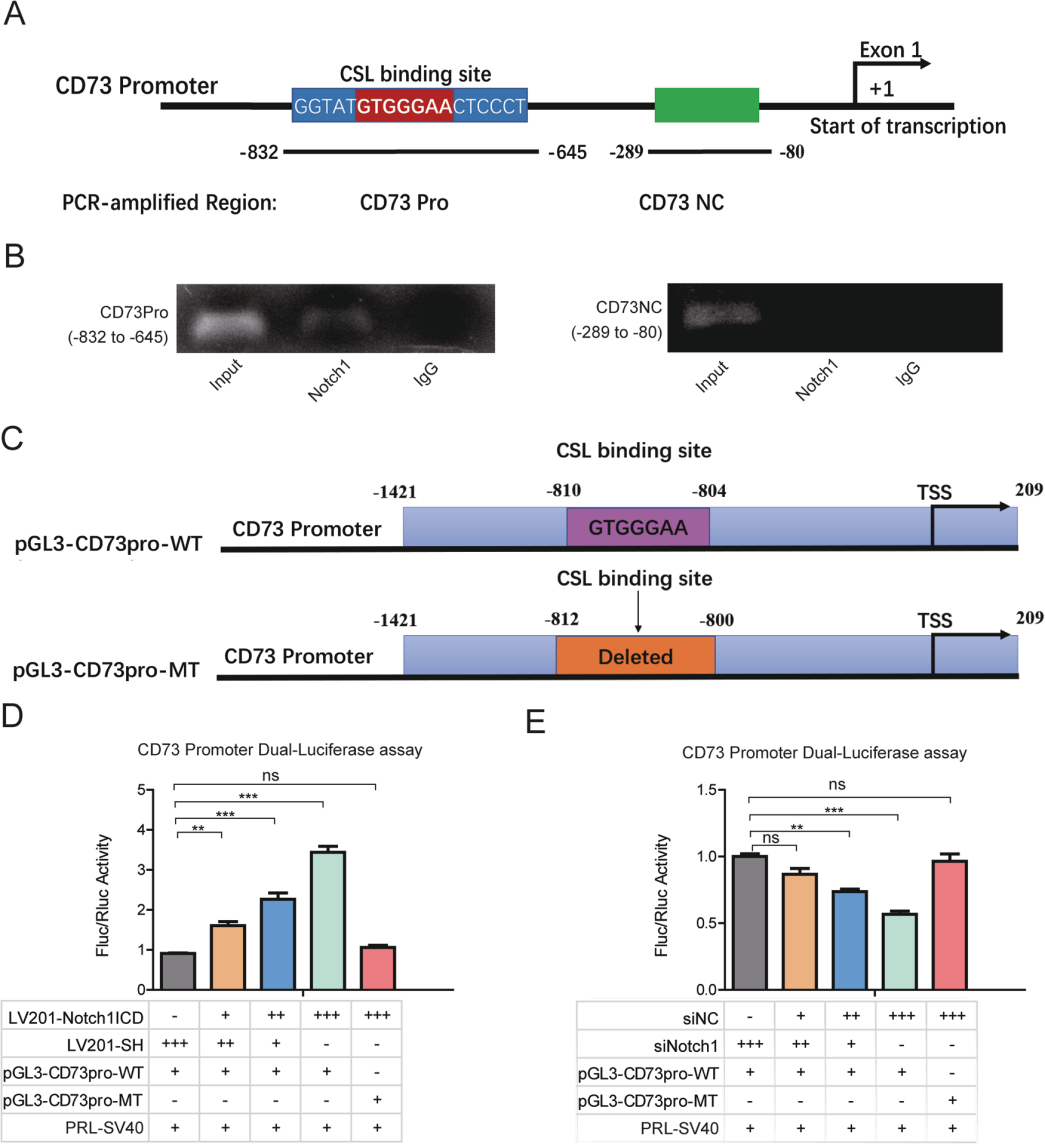
Notch1 upregulates CD73 transcription by binding to its promoter. **A** Graphic representation of the Notch CSL binding site region in the CD73 proximal promoter and Negative control region. **B** The recruitment of Notch1 to the CD73 promoter in MDA-MB-231 cells was assessed using ChIP assays. PCR products were detected in the presence of anti-Notch1 primary antibody. **C** Schematic illustration of the establishment of CD73 promoter luciferase reporter (pGL3-CD73pro-WT) and individual Notch CSL binding site deletion mutant (pGL3-CD73pro-MT) vector. **D** Dual-Luciferase assays were performed in MDA-MB-231 cell by co-transfection with LV201-SH, LV201-N1ICD, pGL3-CD73pro-WT or pGL3-CD73pro-MT and luciferase activity was normalized to the Renilla minimal. **E** Notch1 was silenced in MDA-MB-231 cells by siRNA and then co-transfected with the pGL3-CD73pro-WT or pGL3-CD73pro-MT. Dual-Luciferase values were used to indicate promoter activity. *p < 0.05, **p < 0.01, and ***p < 0.001 (Student’s *t* test) as compared with control cells. Data were presented as the mean ± SEM. (*n* = 3).

Since expression of CD73 appeared to be regulated by Notch1 expression levels, the nucleotide sequence of the promoter of CD73 was evaluated for the known consensus binding sequence of the Notch1 interaction protein CSL. The promoter region of CD73 indeed contained a putative CSL binding core sequence (GTGGGAA) locating at −810–804bp. In a subsequent chromatin immunoprecipitation (ChIP) assay on MDA-MB-231 cells, primary antibody of Notch1 specifically pulled down the sequence containing the notch-specific binding CSL site of CD73 promoter, where as control IgG did not. The Notch1 monoclonal antibody, however, did not bind to a random sequence of the CD73 promoter region that did not contain CSL (Fig. 3A, B).

Subsequently, a dual-luciferase assay was used to demonstrate that Notch1 binds to the CD73 promoter and upregulates promoter activity using a luciferase reporter vector containing the CSL binding sites (pGL3-CD73pro-WT) and a mutant plamid in which the CSL binding site was deleted (pGL3-CD73pro-M) (Fig. 3C). When co-transfected with 100 ng, 200 ng and 400 ng LV201-N1ICD, the activity of CD73 promoter increased in MDA-MB-231 cells in a dose-dependent manner, whereas up-regulation of NICD did not affect the activity of the CD73 promoter with a point mutation in the CSL binding site (Fig. 3D). In addition, downregulation of Notch1 by siRNA decreased its binding to the CD73 promoter in the CSL region, but not in the cells transfected with CD73pro-MT (Fig. 3E).

### CD73 diminished cisplatin response mediated by deficient Notch1

**Fig. 4:**
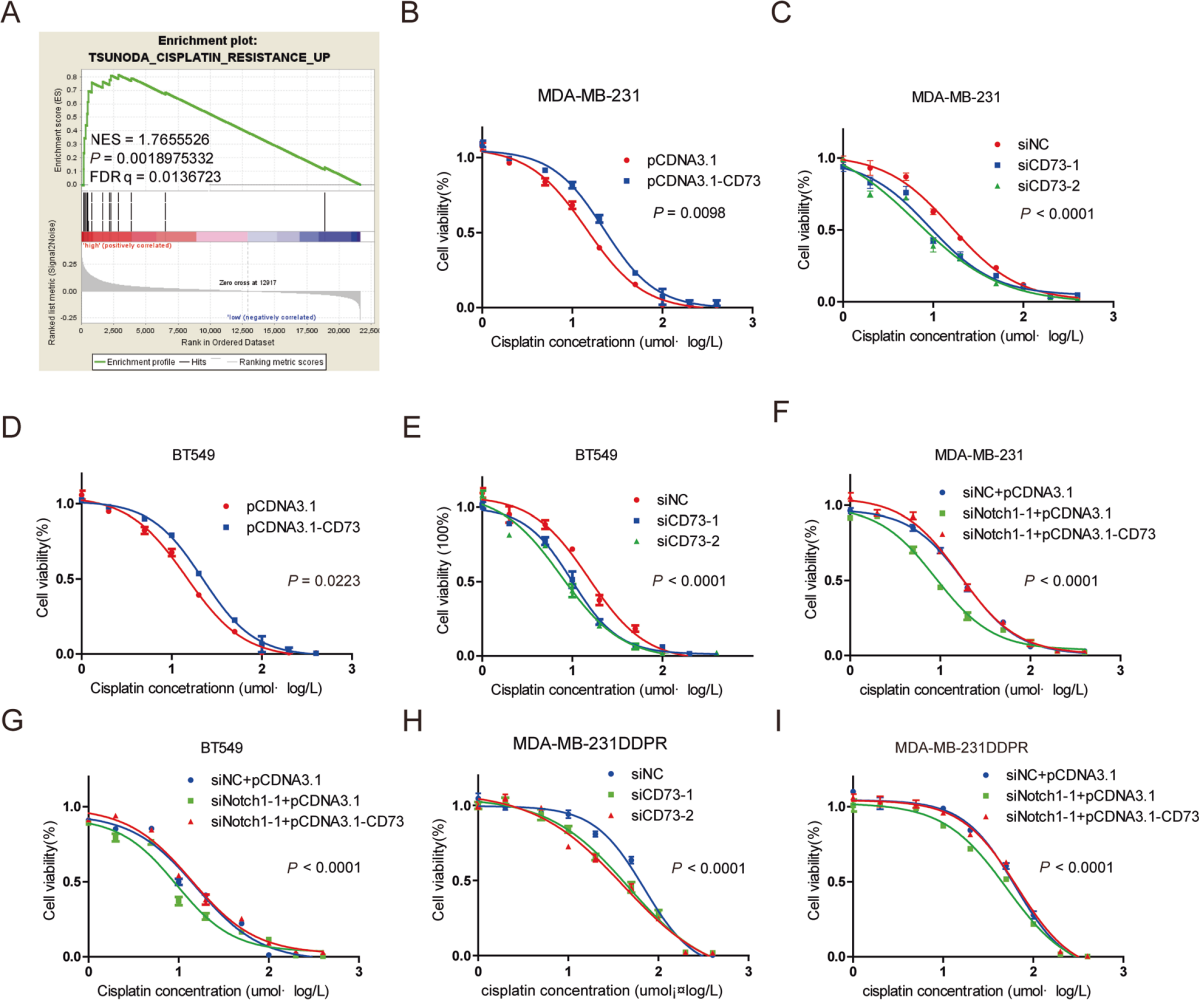
CD73 contribute to cisplatin resistance and diminished cisplatin response mediated by deficient Notch1. **A** CD73 expression positively correlates with cisplatin resistant signaling according to a GSEA plot (GSE76124, *n* = 198). FDR false-discovery rate q value. NES normalized enrichment score. The cell viability analysis following overexpressed CD73 or knock-down CD73 **B**, **C** in MDA-MB-231 cells, **D**, **E** in BT549 cells and (**H**) in MDA-MB-231DDPR cells. **F**, **G**, **I** Rescue assay of cell viability analysis, co-transfected with siNotch1-1 and pCDNA3.1-CD73, 48 h after treatment with cisplatin. Data were presented as the mean ± SEM. of three experiments. *p < 0.05, ** *p* < 0.01 and ***p < 0.001 (Student’s *t* test) as compared with control cells.

GSEA analysis showed that CD73 mRNA level significantly correlated with a cisplatin resistance signature (GSE76124, *n* = 198, *p* = 0.0018975332; Fig. 4A). Notably, when the level of CD73 was increased in MDA-MB-231 cells by transfection with pCDNA3.1-CD73, the IC50 concentration of cisplatin increased from 14.36 ± 1.084 umol/L to 22.48 ± 1.055 umol/L (Fig. 4B). Reversely, knock-down of CD73 by siRNA decreased the IC50 concentration of cisplatin from 16.28 ± 1.017 umol/L to 9.206 ± 0.9480 umol/L (siCD73-1 group) or 6.529 ± 1.133 umol/L (siCD73-2 group), respectively (Fig. 4C). Similar effects of CD73 expression level on cisplatin sensitivity was identified in BT549 cells (Fig. 4D, E). In addition, in the acquired resistant MDA-MB-231DDPR cell line, deletion of CD73 reduced the IC50 of cisplatin by ~60% (Fig. 4H). Thus, CD73 expression is associated with cisplatin resistance.

It is widely accepted that Notch1 plays a role in mediating drug resistance in multiple malignant tumors [24, 25], and our previous study indeed demonstrated that Notch1 increased the resistance to cisplatin in TNBC cells [17]. Since Notch1 directly regulated the expression level of CD73, the potential role of CD73 in Notch1-mediated resistance to cisplatin treatment was evaluated. As expected, knockdown of Notch1 in TNBC cells significantly decreased the IC50 concentration of cisplatin (Fig. 4F, G). However, overexpression of CD73 counteracted the Notch1 depletion-mediated decrease of IC50 concentration (Fig. 4F, G), suggesting CD73 is at least partly regulating the Notch1-reduced cisplatin sensativity in TNBC. Next, the effects of Notch1-CD73 module were examined in acquired resistant MDA-MB-231DDPR cells (Fig. 4I). Again, the IC50 concentration of cisplatin was significantly reduced when Notch1 was depleted, but remained significantly higher upon simultaneous CD73 overexpression (Fig. 4I). Altogether, these data strongly suggest that Notch1-mediated cisplatin resistance by modulating CD73 expression in MDA-MB-231DDPR cells.

## Discussion

**Fig. 5:**
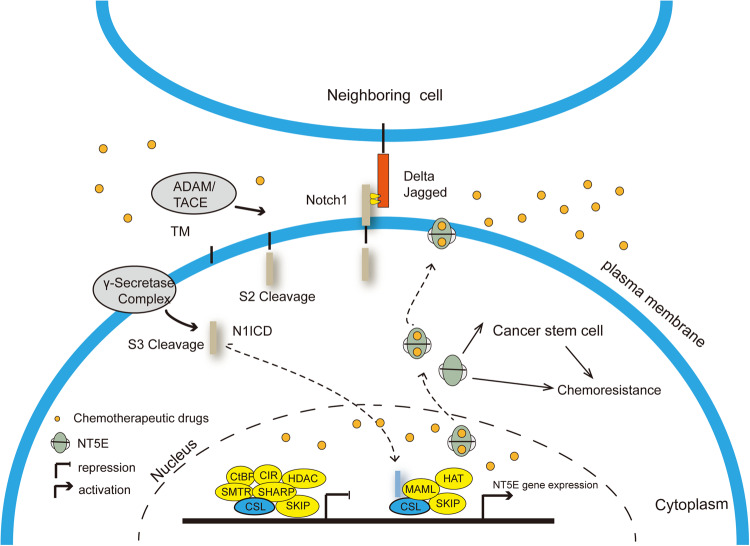
Enhanced Notch1-mediated chemoresistance by transcriptional up-regulating CD73 expression. Notch1 could up-regulate the CD73 expression through binding to the CBF-1 transcription factor.

In this study, we identified Notch1 as a direct regulator of CD73 expression in TNBC. Expression of both Notch1 and CD73 associated with poor pathologic response in patients after four-cycles of cisplatin single treatment and negatively associated with poor RFS in TNBC cases after chemotherapy treatment. In line with this, Notch1 and CD73 were significantly upregulated in MDA-MB-231DDPR, with up or downregulation of CD73 modulating cisplatin sensitivity in TNBC cell lines. Further, Notch1 transcriptionally upregulated CD73 expression by binding to the CSL binding site of its promoter. The results of this study identify CD73 as a novel direct downstream target of Notch1 that is positively correlated with the sensitivity of TNBC cells to cisplatin. This Notch1-CD73 axis may be novel mechanism underlying Notch1-mediated cisplatin resistance (for schematic see Fig. 5).

Our data clearly position CD73 as a factor contributing to resistance to cisplatin in breast cancer. This finding is in line with reports on CD73 in other cancers. For instance, silencing of endogenous CD73 by siRNA enhanced the chemosensitivity in non-small cell lung cancer [26], ovarian cancer and breast cancer [27]. In our study, we found a higher expression of CD73 in acquired cisplatin resistant MDA-MB-231DDPR cell lines than in the susceptible parental cell lines, with deletion of CD73 restoring the sensitivity of MDA-MB-231DDPR cells to cisplatin. This is the first report of the potentiality of silencing CD73 to partly eliminate acquired resistance to cisplatin in TNBC, and is consistent with previously reported results for cisplatin-resistant ovarian cancer cell lines.

CD73 was found to act as a key mediator in the regulation of Notch1-mediated cisplatin resistance in TNBC cells. High Notch1 expression in malignancies is associated with poor prognosis [28] and contribute to intrinsic or secondary drug-resistance to cisplatin [29–31]. The N1ICD directly interacted with the CD73 promoter (at position 810–804 bp) and increased transcriptional activity, leading to upregulation of CD73 at the mRNA and protein level. In addition, dual-fluorescent reporter assays confirmed that N1-ICD drives CD73 promoter activity. The above results validated CD73 is a direct target of Notch1 via a transcriptional regulation in TNBC cells via canonical Notch pathway. Moreover, in rescue assays the absence of Notch1 substantially improved the sensitivity of TNBC cells to cisplatin, but ectopic expression of CD73 largely attenuated this increase. Thus, CD73 is not only an independent factor in the efficiency of cisplatin treatment for TNBC, but it can also function as a direct downstream of Notch1 regulated by the notch classical pathway.

CD73 is also an important enzyme involved in ATP catalysis to adenosine, with adenosine having prominent immunusuppresive effects in the tumor microenvironment. Immunotherapies using CD73 monocular antibody show great potential for immune anti-tumor therapy, and its combination with tartgeing PD1/PD-L1 sigalling achieved a remarkable synergy based on IFN-γ and CD8^+^ T cells [32, 33]. Cisplatin displayed synergistic activity with PD-1 antibody to induces T cells infiltration and secretion of anti-tumor cytokines by activation of CD8 + T cells [34]. Notch1 has also been reported to act as an important mediator of T cell activity [35], but its immune anti-tumor effects have not been elucidated. In this study, we report that the Notch1/CD73 axis plays a role in cellular cisplatin sensitivity, but how it affects the tumor immune microenvironment will need to be further evaluated.

In conclusion, this study identified CD73 as a novel target of an as yet unrecognized molecular mechanism in Notch1-mediated chemoresistance.

## Materials and methods

### Cell culture and establishing the cisplatin-resistant MDA-MB-231DDPR cell line

Human breast cancer cell lines (MDA-MB-231, BT549, M453) and non-TNBC cell lines (T47D, MCF-7) were purchased from American Type Culture Collection (ATCC), routinely cultured in DMEM 10% serum with 1% penicillin/streptomycin in a humidified 5% CO2 incubator at 37 °C. Cisplatin-resistant MDA-MB-231 cells (MDA-MB-231DDPR) were obtained by continuous exposure to increasing dose of cisplatin from 0.1 µg/L to 1 µg/L for at least 6 months. The cell line obtained in this manner, termed MDA-MB-231DDPR, was maintained in routine culture with cisplatin at 1 µg/L.

### Transfection, quantitative real-time PCR and Western blotting

The LV201-SH, LV201-N1ICD, pCDNA3.1 and pCDNA3.1-CD73 plasmids were generated by our laboratory. Small interfering (si) RNAs for knockdowning Notch1 or CD73 were synthesized by GenePharma Company (Suzhou, China). Cancer Cells were plated into cell culture dishes (60 mm diameter) one day in advance, and the confluency of adherent cells should be 40–50% at the time of transfection. Lipofectamine 3000 (Life Technology, NY, USA) was used according to the manufacturer’s protocol for each transfection, then cells were cultivated with antibiotics-free medium. siRNA (500 2pmol) or plasmid (5 ug) was packed in 400 ul Opti-MEM serum medium containing P3000™ Reagent, mixed with dilution in another containing Lipofectamine 3000 (Life Technology, NY, USA) and the mixture was added to the cells after 20 min. RNA and protein were extracted 24 and 48 h after transfection, respectively.

Total mRNA extraction, real-time PCR assay, and Western blot was performed as previously described [36]. The antibodies, primer sequences, and siRNA sequences used are shown respectively in Table 1-1 and Table 1-2.

**Table. 1-1:** Antibody information.

Target	Molecular mass (kDa)	Isotype	Application	Company & Clone#
CD73	73	Mouse IgG	WB(1:3000)	Abcam/([1D7])
			IHC(1:400)	
β-actin	37	Mouse IgG	WB (1:3000)	CST/(8H10D10)
Notch1	120	Rabbit IgG	WB (1:3000)	CST/(C37C7)
			IHC(1:200)	

Tables 1–2	Primers and used Small interference RNAs in this study.		
	Target		5′——3′
siRNA	Notch1-1		5-CACCAGUUUGAAUGGUCAATT-3
	Notch1-2		5-GUGUCUGAGGCCAGCAAGATT-3
	CD73-1		5- TCTCAATCATGCCGCTTTA-3
	CD73-2		5- GCCACTAGCATCTCAAATA-3
qRT-PCR	Notch1	Forward	CGGGTCCACCAGTTTGAATG
		Reverse	GTTGTATTGGTTCGGCACCAT
	CD73	Forward	GGTGGCTTT TAGGAT GGCAAG
		Reverse	ACTGGAACGGTGAAGGTGACAG
	β-actin	Forward	GGTGGCTTTTAGGATGGCAAG
		Reverse	ACTGGAACGGTGAAGGTGACAG
ChIP	CD73 NC	Forward	AAAGCAAAGAAGGGCAGCAT
		Reverse	TGGGTTGAGAACGCACGA
	CD73 pro	Forward	GGCAATATGGGTGATGGGTATGTG
		Reverse	CTTCTGGGACACCCTCTAATGTCATG

### Anti-cancer drug sensitivity assays

MDA-MB-231 cells, BT549 cells and MDA-MB-231DDPRCells were seeded into 96-well plates at a density of 5 × 10^3^ cells/well. Cells at each plate were grown with the different cisplatin concentrations (0, 1, 2, 5, 10, 20, 50,100, 200, and 400 umol/l) for 48 h. Then, 10% CCK-8 was added to each well for 2 h incubation at 37 °C. The absorbance was monitored by at 450 nm using a spectrophotometer (Thermo). And cell viability was represented by IC50 value (median lethal dose) calculated with GraphPad Prism5.

### Chromatin immunoprecipitation (ChIP)

For target validation, we designed two PCR primers (Tables 1–2) flanking the putative Notch1 CSL binding site in the CD73 promoter region, one containing a sequence containing the CSL core sequence (−832 to −645, labeled CD73 Pro) and the other a negative control with GTGGGAA deletion (−289 to −80, labeled CD73 NC). The binding of Notch1 primary antibody to DNA was detected by conventional PCR assay, using the ChIP assay kit (Beyotime, shanghai, China).

**Table 2:** The association between Notch1 and CD73 expression.

Notch1	CD73				Spearman	*P* value
	−	+	++	+++		
−	5	8	3	2	0.33	0.016
+	2	7	3	3		
++	3	4	2	3		
+++	0	1	3	4		

Spearman rank correlation was used to analyze correlation. Two-tailed were statistically tested, *p* < 0.05 has statistical significance.

### Dual-luciferase assay

The CD73 promoter region (−1428 upstream of transcriptional start site and extending to 209 bp) was cloned into the SacI/Smal sites of pGL3-Enhancer vector (Panomics, Fremont, CA, USA) shows the primers used to amplify the target sequence, with one primer containing the CSL binding site (pGL3-CD73pro-WT) and a mutant deleting the CSL binding site (pGL3-CD73pro-MT). MDA-MB-231 cells were seeded in 12-well plates, and co-transfected in a concentration gradient manner with LV201-SH, LV201-N1ICD, siNocth1, siNC, and pRL-SV40 (Promega Madison, WI, USA; E2231). After a total of 48 h of treatment, the luciferase activity was measured by using ONE_Glo EX Dual-Luciferase Assay System kit (Promega; E8110).

### Immunohistochemistry assay and online database analysis

TNBC primary lesions were histopathologically and clinically diagnosed as BC and collected from 53 patients at the Cancer Hospital of Shantou University Medical College between August 2014 and August 2016. The study was approved by the ethical committee of Cancer Hospital of Shantou University. Written informed consents were obtained from patients in accordance with principles expressed in the Declaration of Helsinki. The pathological scoring was carried out by two independent observers.

Array of gene expression profiles (GSE18864) were obtained from a neoadjuvant trial of cisplatin monotherapy in TNBC patients at stage II or III, and tumor response was assessed by the Miller-Payne scoring system. Patients are grouped with good pathologic responses (Miller-Payne score of 3, 4, or 5), hereafter termed a pathologic complete response (pCR), and poor pathologic responses (progressive, Miller-Payne score of 1,2) [6]

Three genomic profiles of breast cancer (*n* = 4421), including GSE81538, GSE96058, and the Cancer Genome Atlas (TCGA, 2012), contain Notch1 and CD73 expression data by RNA-seq in bc-GenExMiner v4.8 platfrom. Pearson pairwise correlation analysis was applied and plotted for gene correlation targeted analysis between Notch1 and CD73 into a liner relationship. Further, the database divides all patients into TNBC molecular subtype and non-TNBC subtype according to the IHC assessmentshowing the distribution of Notch1/CD73 expression. Microarray data GSE76124 (Platform GPL570) were driven from 198 TNBC patients in GEO database (http://www.ncbi.nlm.nih.gov/geo/) and subjected to Gene set enrichment analysis using GSEA software (version 2.0.13).

To demonstrate the association between the gene expression and prognosis, in UCSC Xeua platfrom, survival probabilities are described in Kaplan–Meier plot based on IlluminaHiSeq data driven from TCGA Breast Cancer (BRCA). Moreover, recurrence-free survival (RFS) of two groups by mRNA levels of CD73 or Notch1, in basal-like patients with chemothepapeutic systemic treatment, was accessed by Kaplan–Meier plotter (http://kmplot.com) (*n* = 230).

### Statistical analysis

Statistical differences were assessed by using SPSS Software (23.0 version) on the mean and SEM in all implemented experiments. Student’s *t* test and unpaired two-tailed Student’s *t* test were used for between-group and paired comparison. Statistical significance was defined as a two-sided *p* value <0.05.

## Supplementary information

supplements

supplementary WB

Original Data File

Original Data File
