# Supplementary material for: Notch1 promotes resistance to cisplatin by up-regulating Ecto-5′-nucleotidase (CD73) in triple-negative breast cancer cells
Source: Cell Death Discov. 2023 Jun 30;9:204. doi: 10.1038/s41420-023-01487-x (PMC10313677; doi:10.1038/s41420-023-01487-x)
Supplement: Supplementary file 1 — supplements [file 41420_2023_1487_MOESM1_ESM.docx]

**
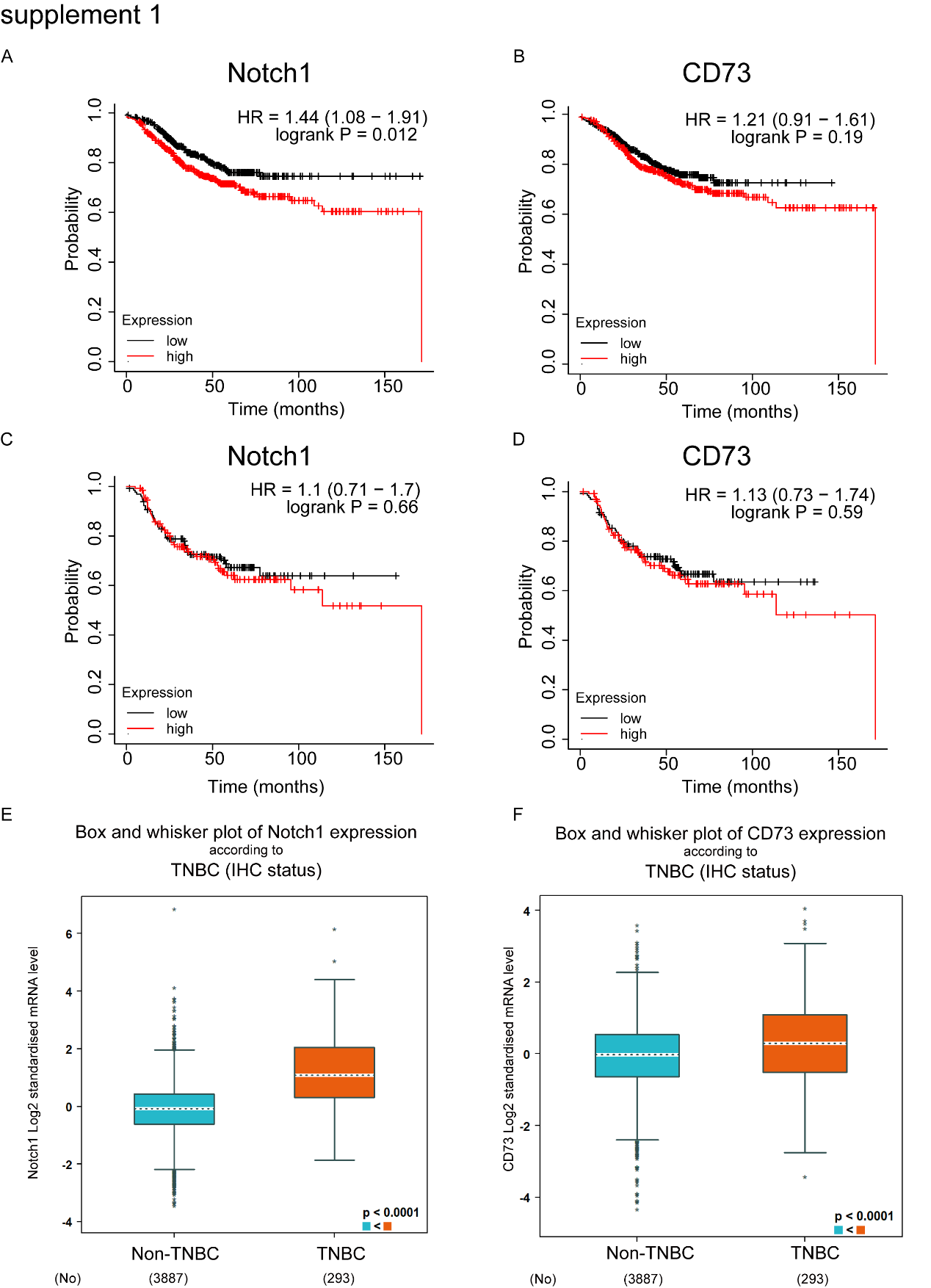
**

**(A-B)**. RFS analysis of Notch1 and CD73 expression level in ER+ breast cancer patients with chemotherapy. **(C-D)**. RFS analysis of Notch1 and CD73 expression level in Her2+ breast cancer patients with adjuvant chemotherapy. **(E-F)** the mRNA levels of Notch1 and CD73 in TNBC subtype tumors and non-TNBC subtype counterparts.
