# Supplementary figures and images for: Notch1 promotes resistance to cisplatin by up-regulating Ecto-5′-nucleotidase (CD73) in triple-negative breast cancer cells
Source: Cell Death Discov. 2023 Jun 30;9:204. doi: 10.1038/s41420-023-01487-x (PMC10313677; doi:10.1038/s41420-023-01487-x)

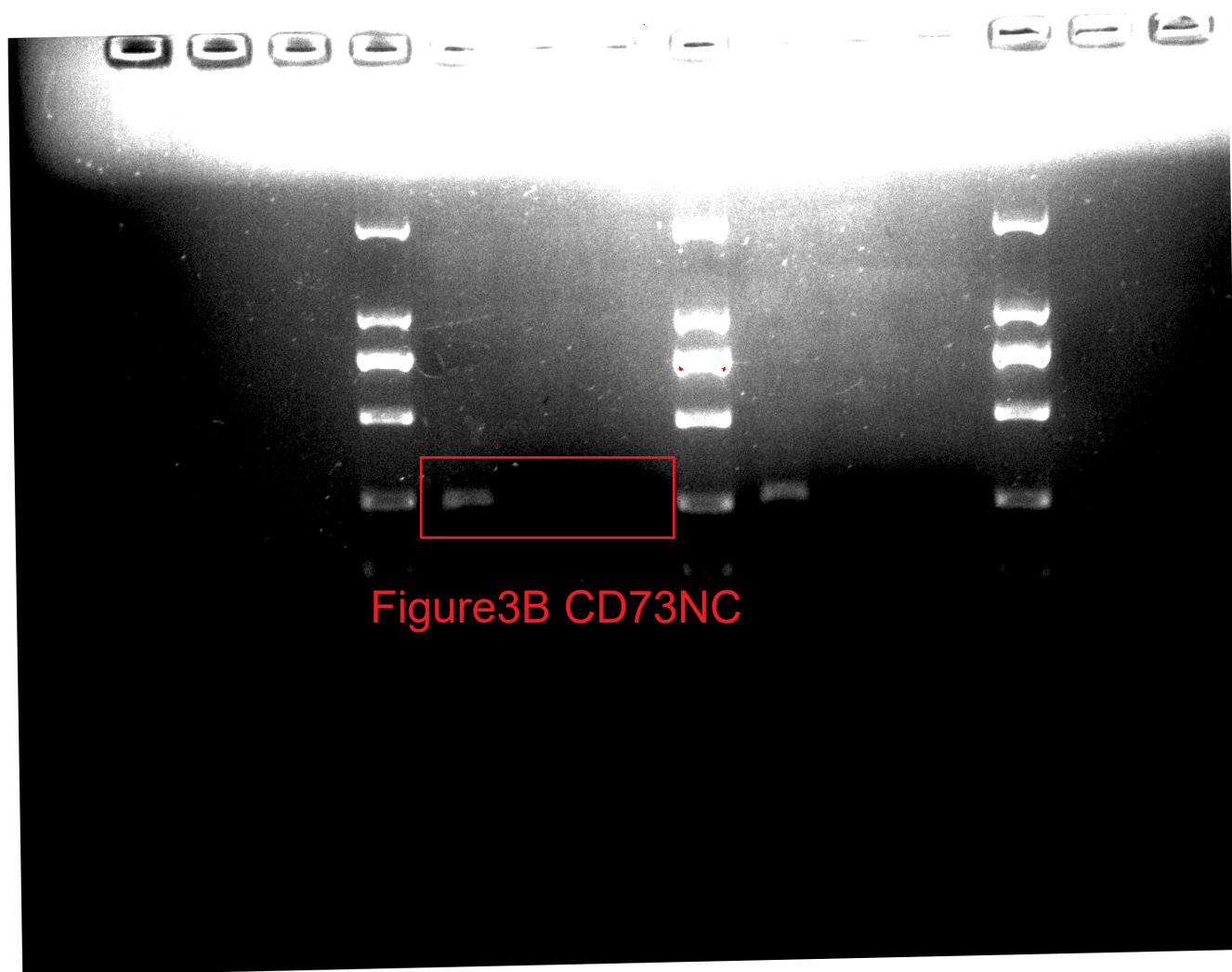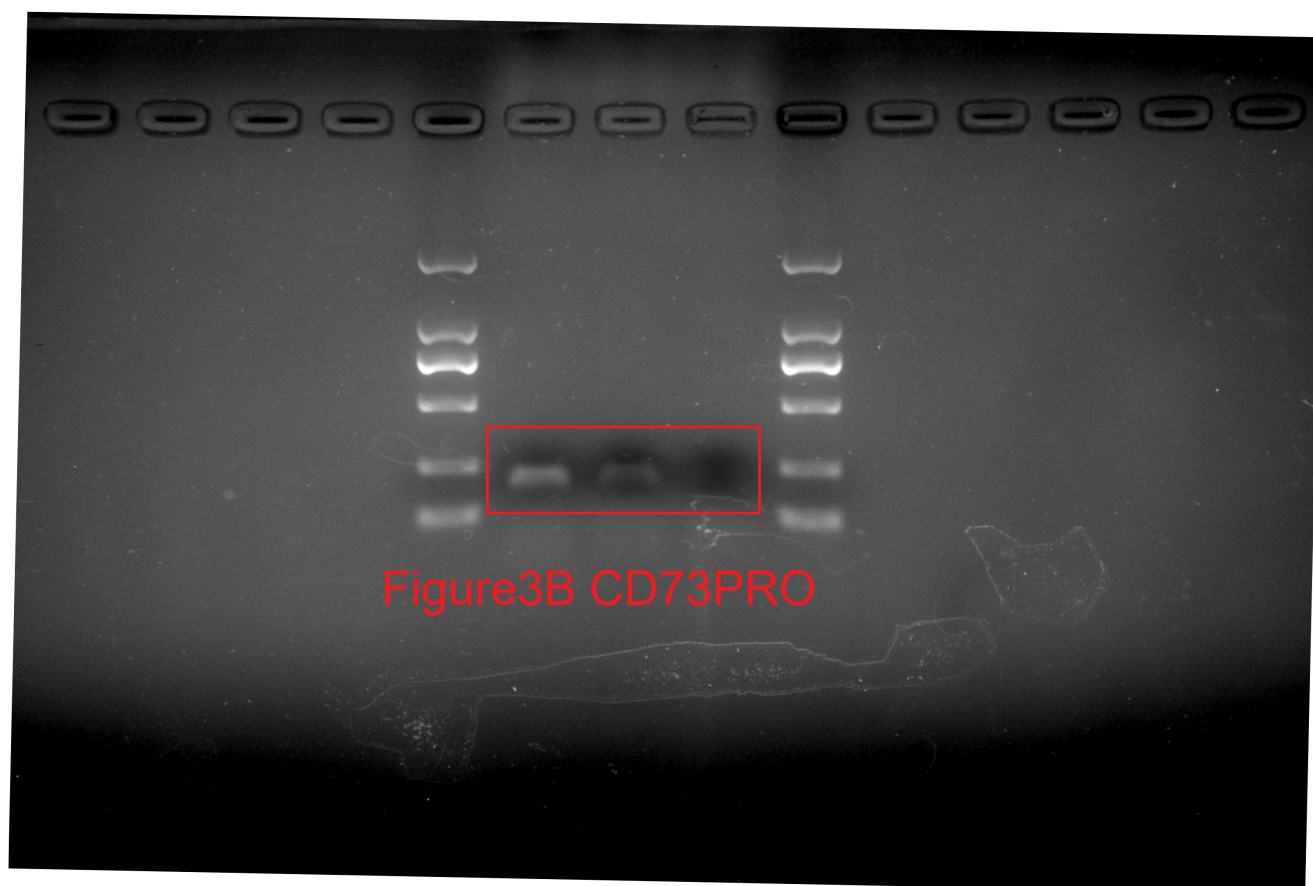

Supplement: Supplementary file 3 — Original Data File [file 41420_2023_1487_MOESM3_ESM.pdf]

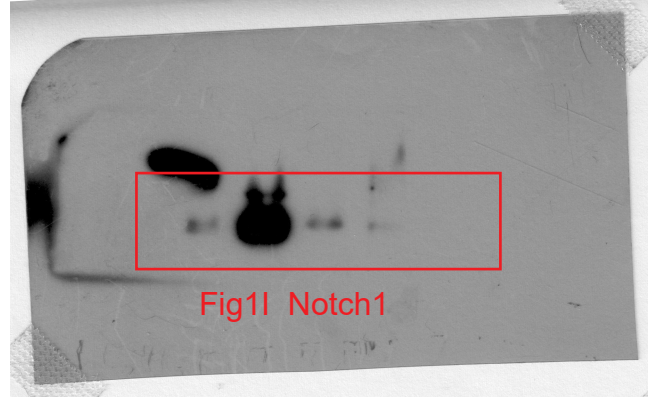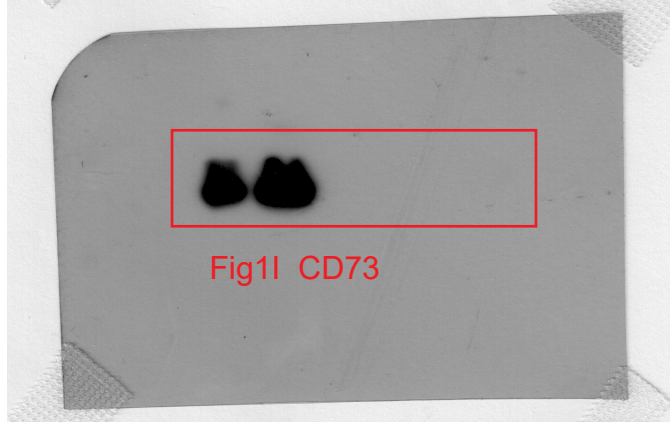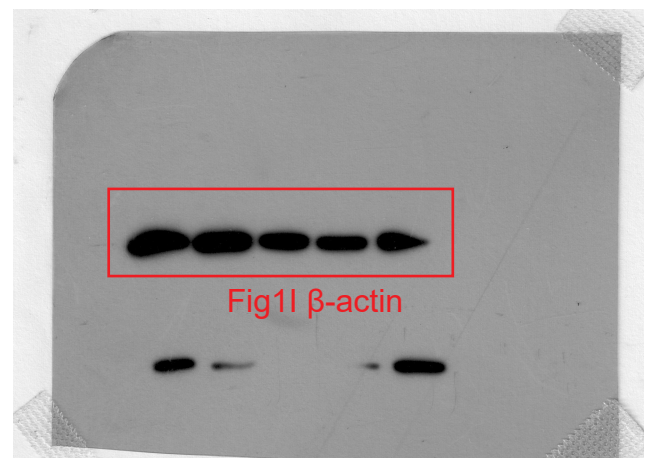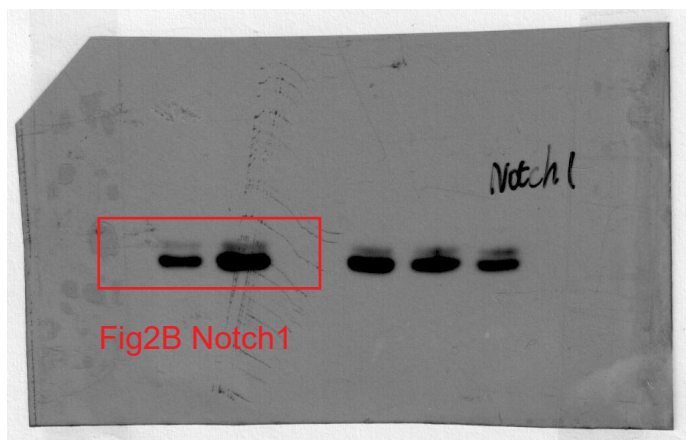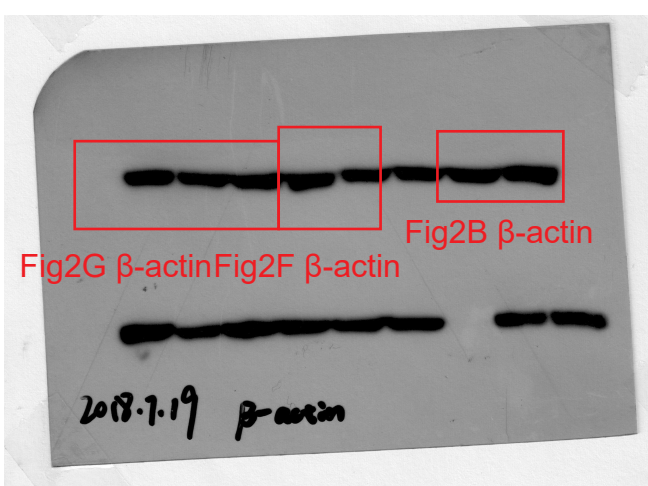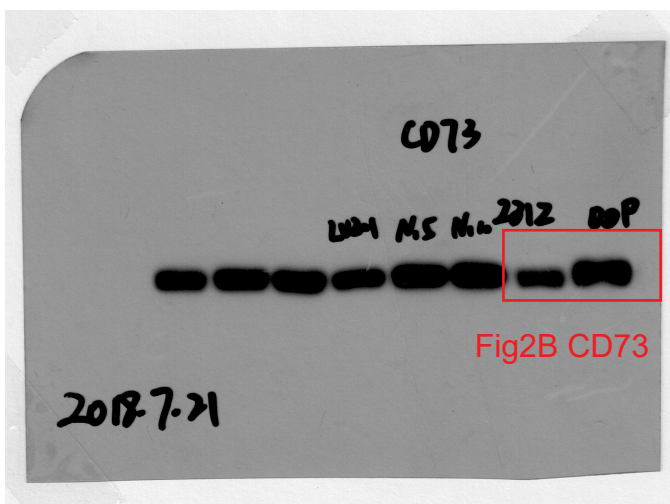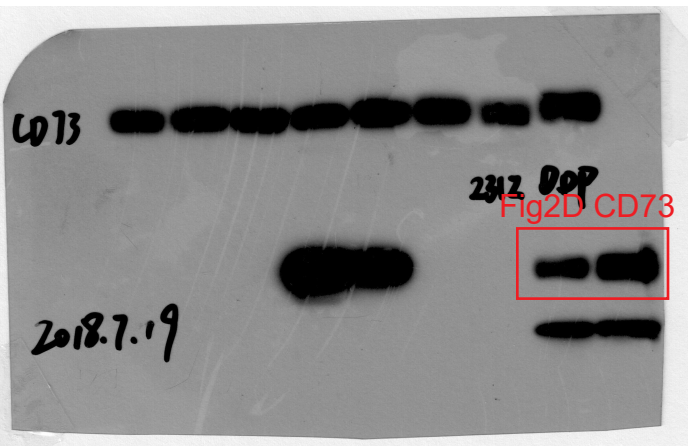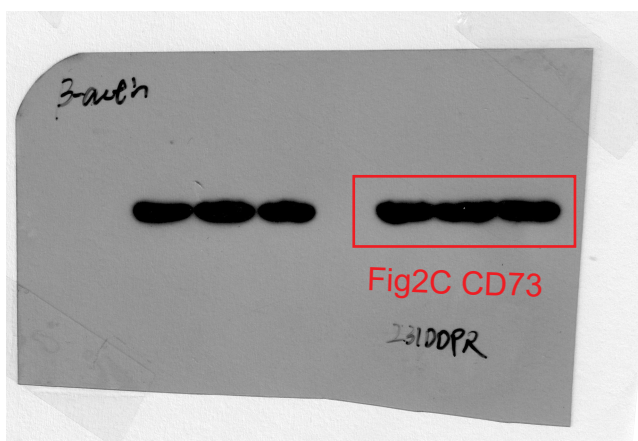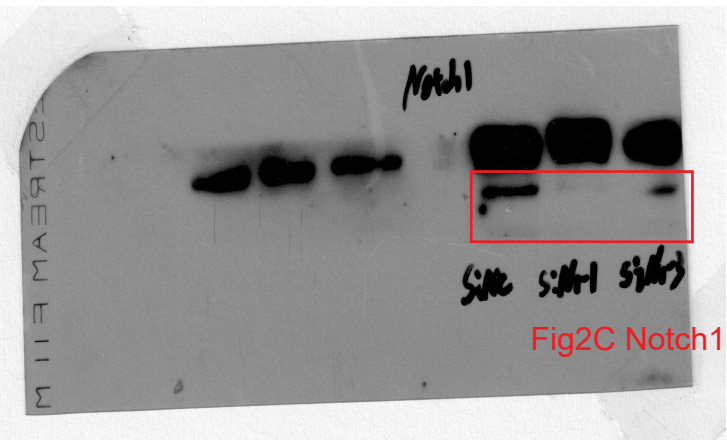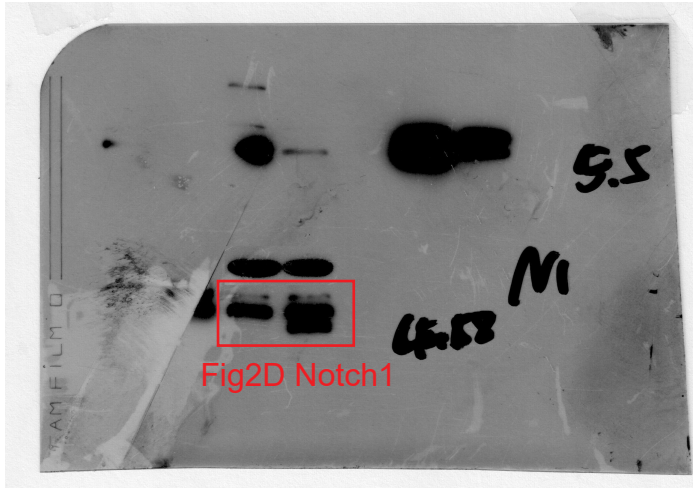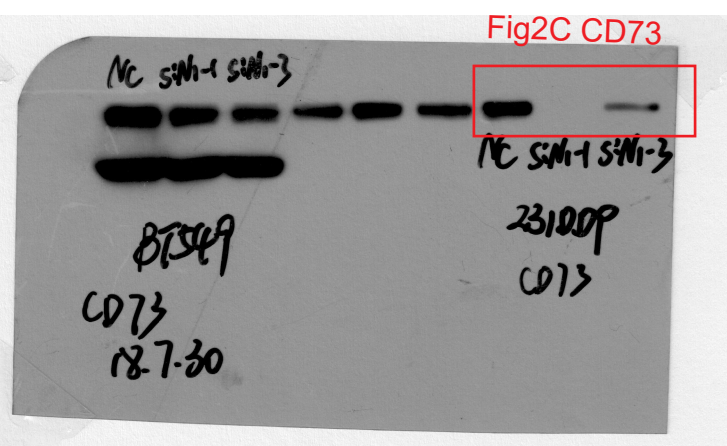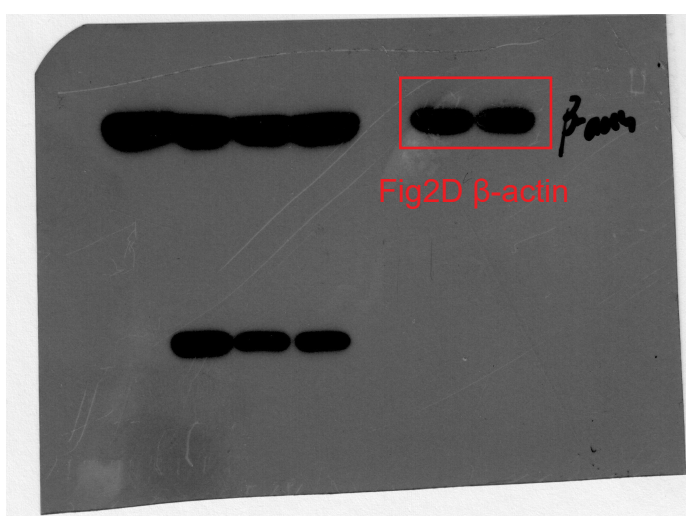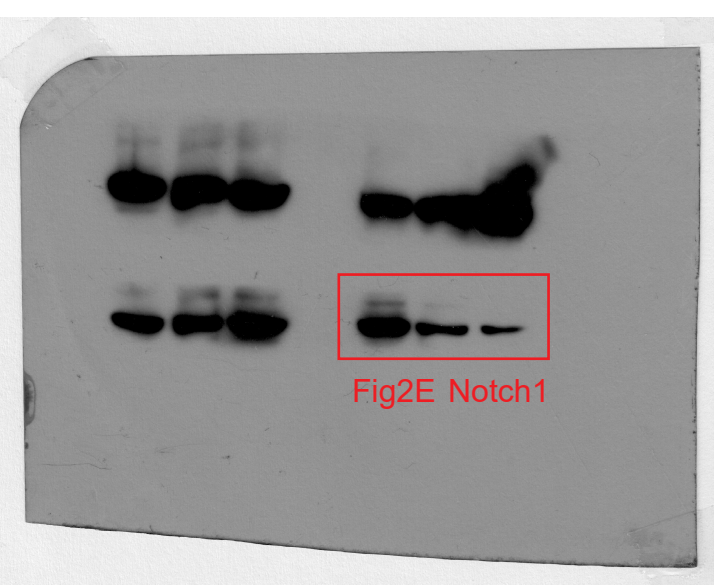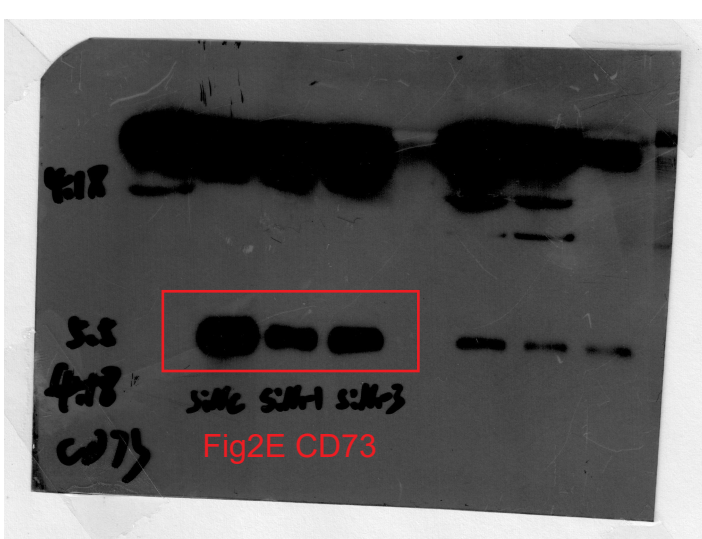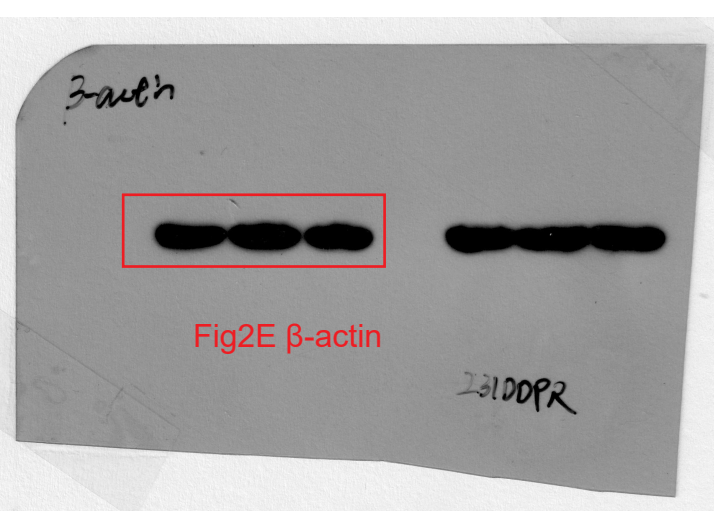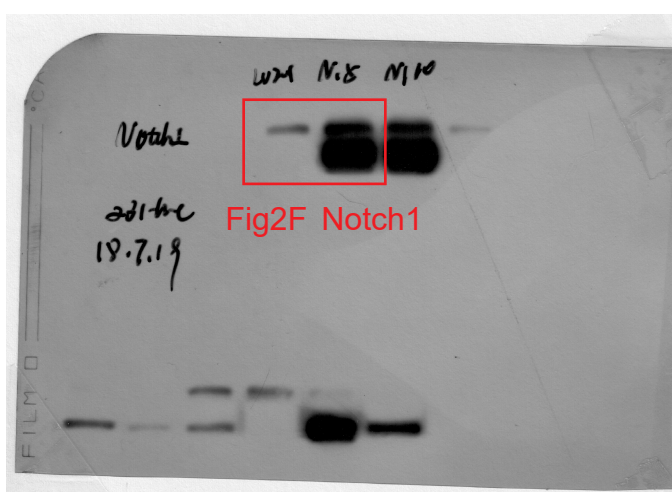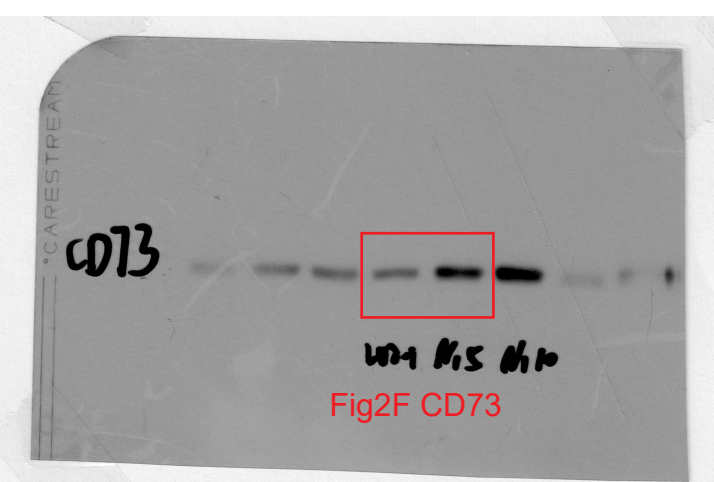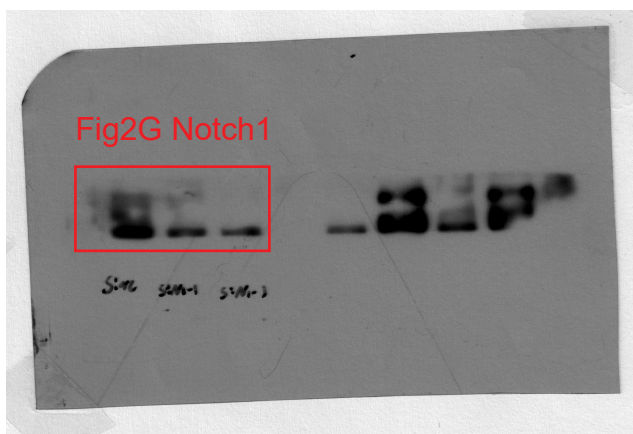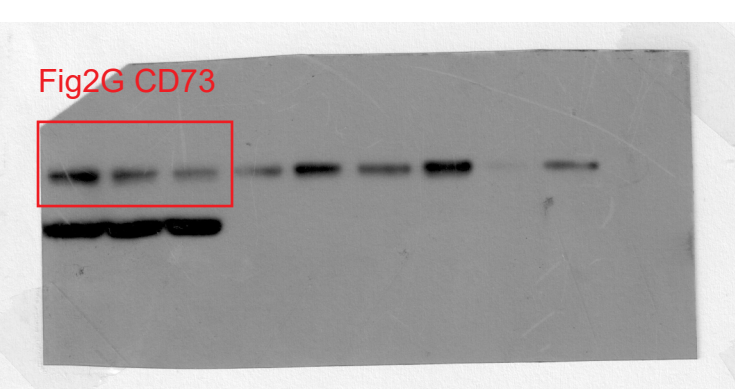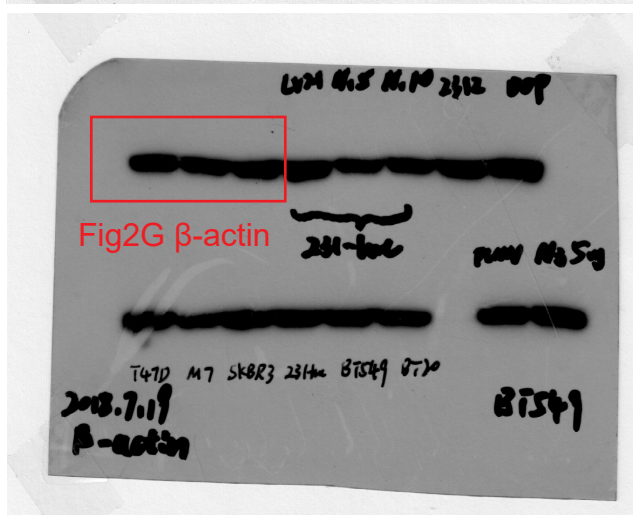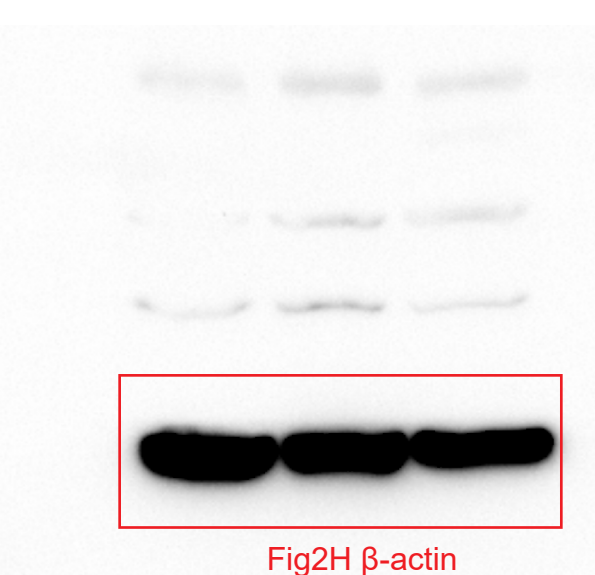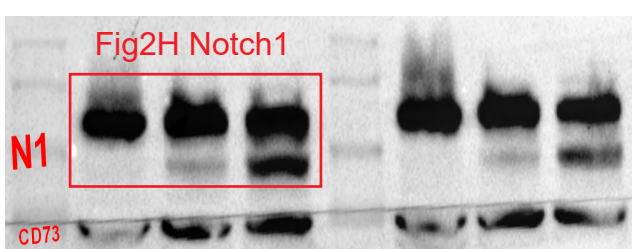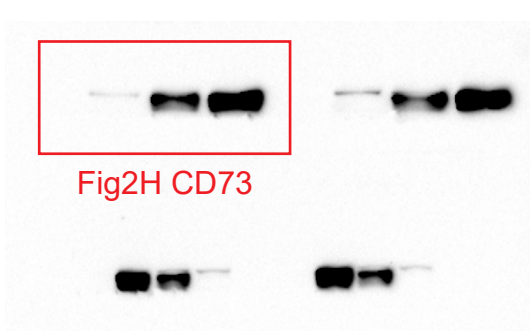

Supplement: Supplementary file 4 — Original Data File [file 41420_2023_1487_MOESM4_ESM.pdf]
