# Supplementary material for: Notch1 promotes resistance to cisplatin by up-regulating Ecto-5′-nucleotidase (CD73) in triple-negative breast cancer cells
Source: Cell Death Discov. 2023 Jun 30;9:204. doi: 10.1038/s41420-023-01487-x (PMC10313677; doi:10.1038/s41420-023-01487-x)
Supplement: Supplementary file 2 — supplementary WB [file 41420_2023_1487_MOESM2_ESM.pdf]

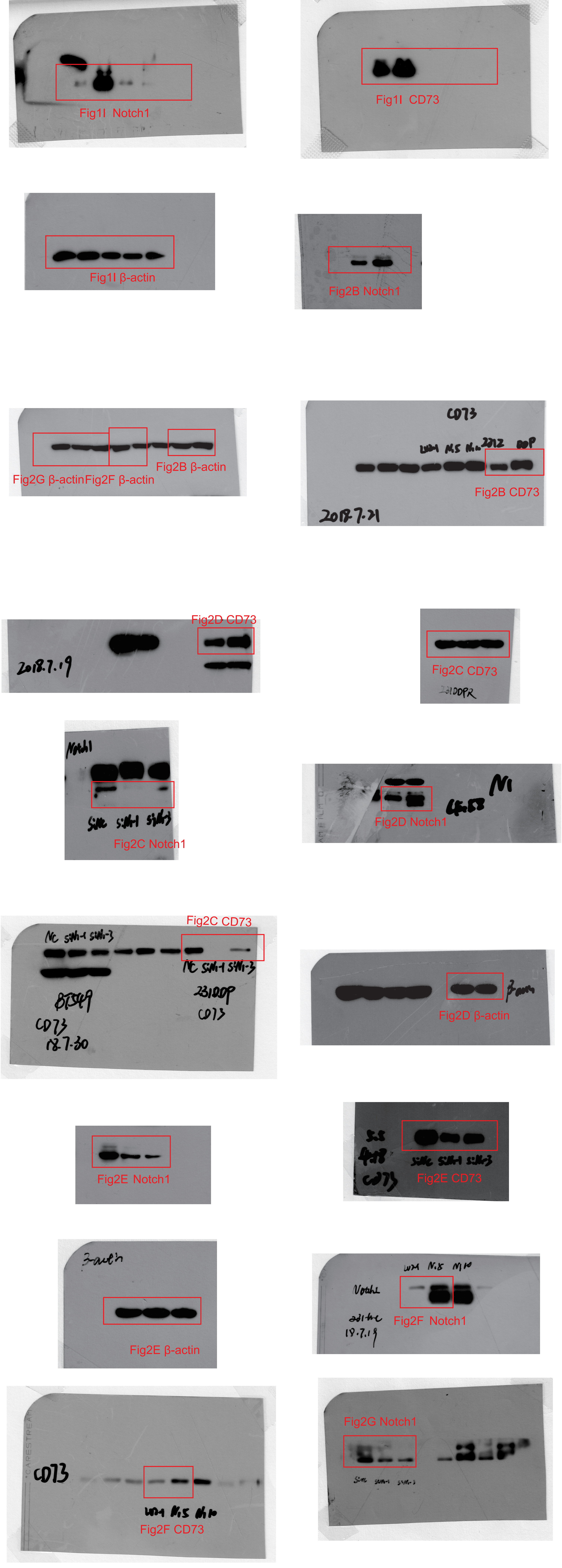
Fig1I Notch1
Fig1I CD73
Fig1I β-actin
Fig2B Notch1
Fig2G β-actin
Fig2F β-actin
Fig2B β-actin
Fig2B CD73
Fig2D CD73
Fig2C CD73
Fig2C Notch1
Fig2D Notch1
Fig2C CD73
Fig2D β-actin
Fig2E Notch1
Fig2E CD73
Fig2E β-actin
Fig2F Notch1
Fig2F CD73
Fig2G Notch1
